# The Small Heat Shock Protein p26 Aids Development of Encysting *Artemia* Embryos, Prevents Spontaneous Diapause Termination and Protects against Stress

**DOI:** 10.1371/journal.pone.0043723

**Published:** 2012-08-27

**Authors:** Allison M. King, Thomas H. MacRae

**Affiliations:** Department of Biology, Dalhousie University, Halifax, Nova Scotia, Canada; Sars International Centre for Marine Molecular Biology, Norway

## Abstract

*Artemia franciscana* embryos enter diapause as encysted gastrulae, a physiological state of metabolic dormancy and enhanced stress resistance. The objective of this study was to use RNAi to investigate the function of p26, an abundant, diapause-specific small heat shock protein, in the development and behavior of encysted *Artemia* embryos (cysts). RNAi methodology was developed where injection of *Artemia* females with dsRNA specifically eliminated p26 from cysts. p26 mRNA and protein knock down were, respectively, confirmed by RT-PCR and immuno-probing of western blots. ArHsp21 and ArHsp22, diapause-related small heat shock proteins in *Artemia* cysts sharing a conserved α-crystallin domain with p26, were unaffected by injection of females with dsRNA for p26, demonstrating the specificity of protein knock down. Elimination of p26 delayed cyst release from females demonstrating that this molecular chaperone influences the development of diapause-destined embryos. Although development was slowed the metabolic activities of cysts either containing or lacking p26 were similar. p26 inhibited diapause termination after prolonged incubation of cysts in sea water perhaps by a direct effect on termination or indirectly because p26 is necessary for the preservation of diapause maintenance. Cyst diapause was however, terminated by desiccation and freezing, a procedure leading to high mortality within cyst populations lacking p26 and indicating the protein is required for stress tolerance. Cysts lacking p26 were also less resistant to heat shock. This is the first in vivo study to show that knock down of a small heat shock protein slows the development of diapause-destined embryos, suggesting a role for p26 in the developmental process. The same small heat shock protein prevents spontaneous termination of diapause and provides stress protection to encysted embryos.

## Introduction

Embryos of the crustacean *Artemia franciscana* develop ovoviviparously, yielding swimming nauplii upon release from females, or they develop oviparously, producing encysted gastrulae known as cysts [1], [2]. *Artemia* cysts enter diapause, a phylogenetically widespread state of dormancy divided into several potentially overlapping phases including initiation, maintenance and termination [3]–[7]. In *Artemia* cysts diapause is distinguished by metabolic activity so low it is difficult to detect experimentally [8], [9] and by tolerance to severe stressors such as repeated freezing and thawing, high salinity, heat and anoxia, the latter for several years [8]–[10]. Diapause termination occurs in response to discrete cues [11], [12] and if hydration, temperature, and aeration are favorable, development resumes. If conditions are unfavorable activated cysts enter a dormant state termed quiescence where they remain until environmental circumstances permit growth.

Molecular chaperones synthesized in diapause-destined embryos of *Artemia*, and which potentially contribute to cyst stress tolerance, include the small heat shock proteins (sHSPs) p26, ArHsp21 and ArHsp22 [1], [13]–[17], as well as artemin, a ferritin homologue [18], [19]. p26, the focus of this report, is an abundant diapause-specific protein [20]. Like other sHSPs [21]–[23], a p26 monomer is composed of an amino-terminal region, the conserved α-crystallin domain and a carboxyl-terminal extension [24], [25], and the monomers associate to form oligomers [14], [26]. The sHSP α-crystallin domain contributes to dimerization, oligomerization and chaperone activity [21]–[23], [27], [28]. In isolation from the remainder of the protein the chaperone activity of the α-crystallin domain is, however, low [29] and dimer formation, at least by yeast Hsp26, requires residues exterior to the α-crystallin domain [30]. The sHSP amino-terminus is involved in subunit dynamics, oligomerization and substrate binding while the flexible carboxyl-terminal extension, characterized by the I/V-X-V/I motif, modulates oligomerization, chaperoning and solubility [22], [23], [26], [31], [32].

The synthesis of p26 is developmentally regulated and expression of the p26 gene is not induced by stress [1], [13], [17]. p26 mRNA first appears in diapause-destined *Artemia* embryos at 2 days post-fertilization and increases until cyst release from females. The p26 protein, detectable only in diapause-destined embryos of *Artemia*, is visible at day 3 post-fertilization and increases in amount until cyst release from females [1], [20]. p26 mRNA and protein are degraded upon diapause termination and resumption of embryo development, and the protein is not observed in embryos developing directly into nauplii, indicating diapause-specific functions. p26 translocates to nuclei during embryo diapause, anoxia, high temperature and low pH [10], [13], [14], [20], [23], [24], [33]. These findings suggest that the protein is multifunctional and has roles within both the cytosol and nucleus.

p26 behaves like a molecular chaperone, protecting proteins against denaturation in in vitro turbidimetric assays [14], [25], [34], conferring stress tolerance on transformed bacteria [25], [34] and transfected mammalian cells [29], [35], and inhibiting apoptosis [29], [35]. These observations imply that p26 binds proteins structurally perturbed by stress, preventing their irreversible denaturation, and that it influences processes such as signaling pathways thus influencing cell activities. During post-diapause development proteins may be released from p26 and folded, either spontaneously or with assistance from ATP-dependent molecular chaperones such as HSP60, HSP70 and HSP90. The refolded proteins are then available for use in growing cells. p26 thereby modulates cell activities during diapause and is likely to contribute to the high stress tolerance of *Artemia* cysts.

In this study double-strand RNA (dsRNA) complementary to p26 was injected into the eggs sacs of adult *Artemia* females prior to fertilization, resulting in extensive knock down of p26 mRNA and the removal of p26 from cysts. Embryos lacking p26 developed more slowly than those with normal amounts of the protein, although cysts arising from both types of embryos displayed equal metabolic activity. Cysts deficient in p26 terminated diapause more readily than p26-containing cysts and exhibited reduced survival upon exposure to different stressors such as desiccation, cold and heat. The results provide an in vivo demonstration of p26 functions during *Artemia* diapause and expand the functions potentially attributable to sHSPs during diapause in all organisms.

## Methods

### Culture of *Artemia*

*A. franciscana* cysts from the Great Salt Lake (INVE Aquaculture, Inc., Ogden, UT, USA) were incubated at room temperature with moderate to strong aeration for 48 h in filtered, autoclaved sea water from Halifax Harbor. Cultures were then incubated without aeration for 30 min, cyst shells were removed from the surface, and harvested larvae were incubated at room temperature with aeration in sea water. *Artemia* were maintained on *Isochrysis sp* (clone synonym TISO) (Provasoli-Guillard Center for Culture of Marine Phytoplankton, West Boothbay Harbor, ME, USA).

**Figure 1:**
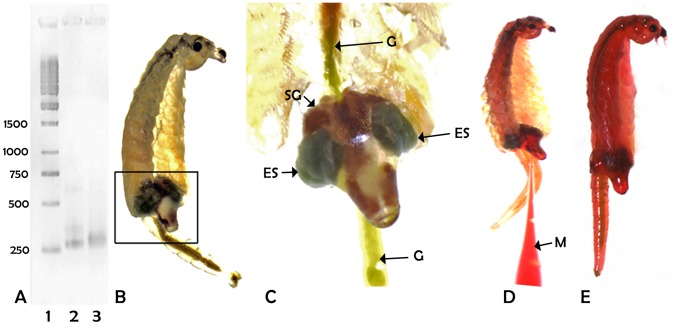
Production of p26 dsRNA and injection of *Artemia* females. A. The production of p26 dsRNA was as described in Methods. Amplification products were resolved by electrophoresis in 1.2% agarose gels and stained with Gelstar®. Lane 1, size markers in bp; 2, p26 cDNA; 3, p26 dsRNA. B–E. Light micrographs showing the injection of a female primed to produce cysts; all images are of the same animal; B. *Artemia* female prior to fertilization with the shell gland and egg sacs boxed; C. Boxed region of Figure B enlarged; D. Injection of dsRNA into the egg sac; E. An injected female which has retained phenol red for 2 h. ES, egg sac; G, gut; M, micropipette; SG, shell gland.

### Preparation of dsRNA

pRSETC plasmids (Invitrogen, Burlington, ON., Canada) containing p26 cDNA excised from pRSETC-p26-3-6-3 [24] were collected from 5 ml overnight cultures of BL21(DE3)pLysS *E. coli* (Invitrogen) using a miniprep kit (Sigma-Aldrich, Oakville, ON., Canada). The DNA encoding p26 was amplified using forward (5′-TAATACGACTCACTAT AGGGAGACCACTCCCAGAACATGTCAAACCA-3′) and reverse (5′-TAATACGACTCA CTATAGGGAGACCACTGCACCTCCTGATCTTGTTG-3′) primers, each specific for p26 and containing the T7 promoter, TAATACGACTCACTATAGG, at the 5′-end (Integrated DNA Technologies, Coralville, IA, USA). The PCR reaction was performed, according to manufacturer’s instructions with 2 units of Platinum® Taq DNA polymerase (Invitrogen) and 0.35 µM of each primer. Amplification was as follows: 5 min at 94°C, 30 cycles at 94°C for 30 sec, 59°C for 30 sec and 72°C for 1 min, followed by 10 min at 72°C. The PCR products were revealed by electrophoresis in 1.5% agarose gels in 0.5×TBE buffer (20 mM TRIS, 20 mM acetic acid, 1 mM EDTA, pH 8.5), staining with Gelstar® (Lonza, Basel, Switzerland) and visualization with a DNR Bio-Imaging Systems MF - ChemiBIS 3.2 gel documentation system (Montreal Biotech, Montreal, QC, Canada). The PCR product was used as template to generate dsRNA using the MEGAscript® RNAi kit (Ambion Applied Biosystems, Austin, TX, USA) according to manufacturer’s instructions. The dsRNA was visualized after agarose gel electrophoresis as described above. The Fermentas 1 kb DNA Ladder (Fermentas Canada Inc., Burlington, ON, Canada) was used as size marker.

**Figure 2:**
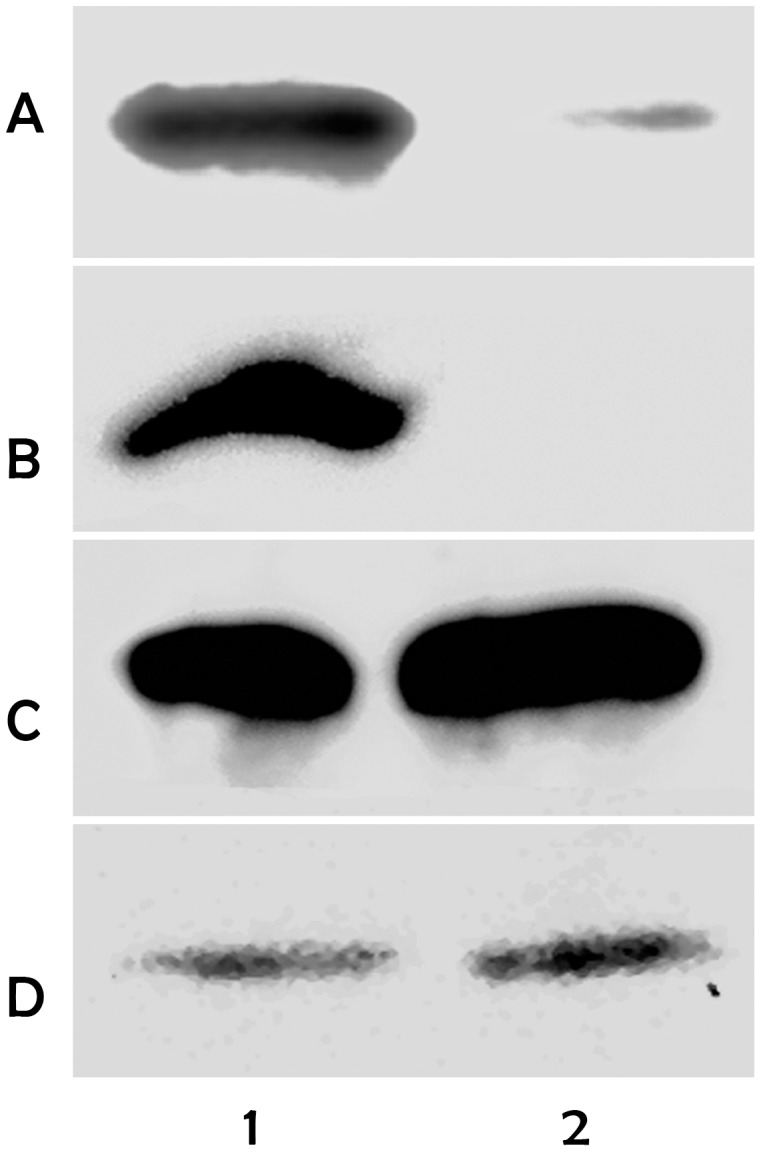
p26 dsRNA specifically knocks down p26 mRNA and protein in *Artemia* cysts. A. PCR amplification of p26 mRNA in cysts released by females injected with either control solution (1) or p26 dsRNA (2). Amplification products were resolved in 1.2% agarose gels and stained with Sybersafe. Protein from 25 cysts (B) and 40 cysts (C, D) produced by females injected with either control solution (1) or p26 dsRNA (2) were resolved in 12.5% SDS polyacrylamide gels and blotted to nitrocellulose. The blots were probed with antibody specific to p26 (B), ArHsp21 (C) and ArHsp22 (D), followed by HRP-conjugated goat anti-rabbit IgG antibody. Antibody reactive proteins were visualized by chemiluminescence.

### Injection of *Artemia* with dsRNA

While observed with an Olympus SZ61 stereomicroscope (Olympus Canada Inc., Markham, ON, Canada) adult *Artemia* females immobilized on 3% agarose cooled to 4°C [36] were injected into the egg sac with 250 nl of p26 dsRNA solution consisting of 80 ng of dsRNA in elution buffer from the MEGAscript® RNAi kit (Ambion Applied Biosystems) mixed in 1∶1 (v/v) ratio with 0.5% phenol red in Dulbecco’s phosphate buffered saline (DPBS) (Sigma-Aldrich). *Artemia* females also received elution buffer mixed in 1∶1 (v/v) ratio with 0.5% phenol red in DPBS (control solution) and green fluorescent protein (GFP) dsRNA in elution buffer mixed in 1∶1 (v/v) ratio with 0.5% phenol red as above. The GFP cDNA used as template for preparation of dsRNA was cloned in pEGFP-N1 (Clonetech, Mountain View, CA, USA). Primers for GFP were forward (5′-TAATACGACTCACTAAGGGAGACACATGAAGCAGC ACGACCT-3′) and reverse (5′-TAATACGACTCACTATAGGGAGAAGTTCACCTTGATG CCGTTC-3′). PCR conditions were as shown previously except the annealing temperature was 60°C and 2.25 mM MgCl_2_ was added to the reaction mixture. Injection was with a Nanoject II microinjector (Drummond Scientific Co., Broomall, PA, USA) using a micropipette prepared with preset program 33 on a P-97 Flaming/Brown Micropipette Puller (Sutter Instrument Co., Novato, CA, USA) and broken on an angle with forceps under a dissecting microscope.

**Figure 3:**
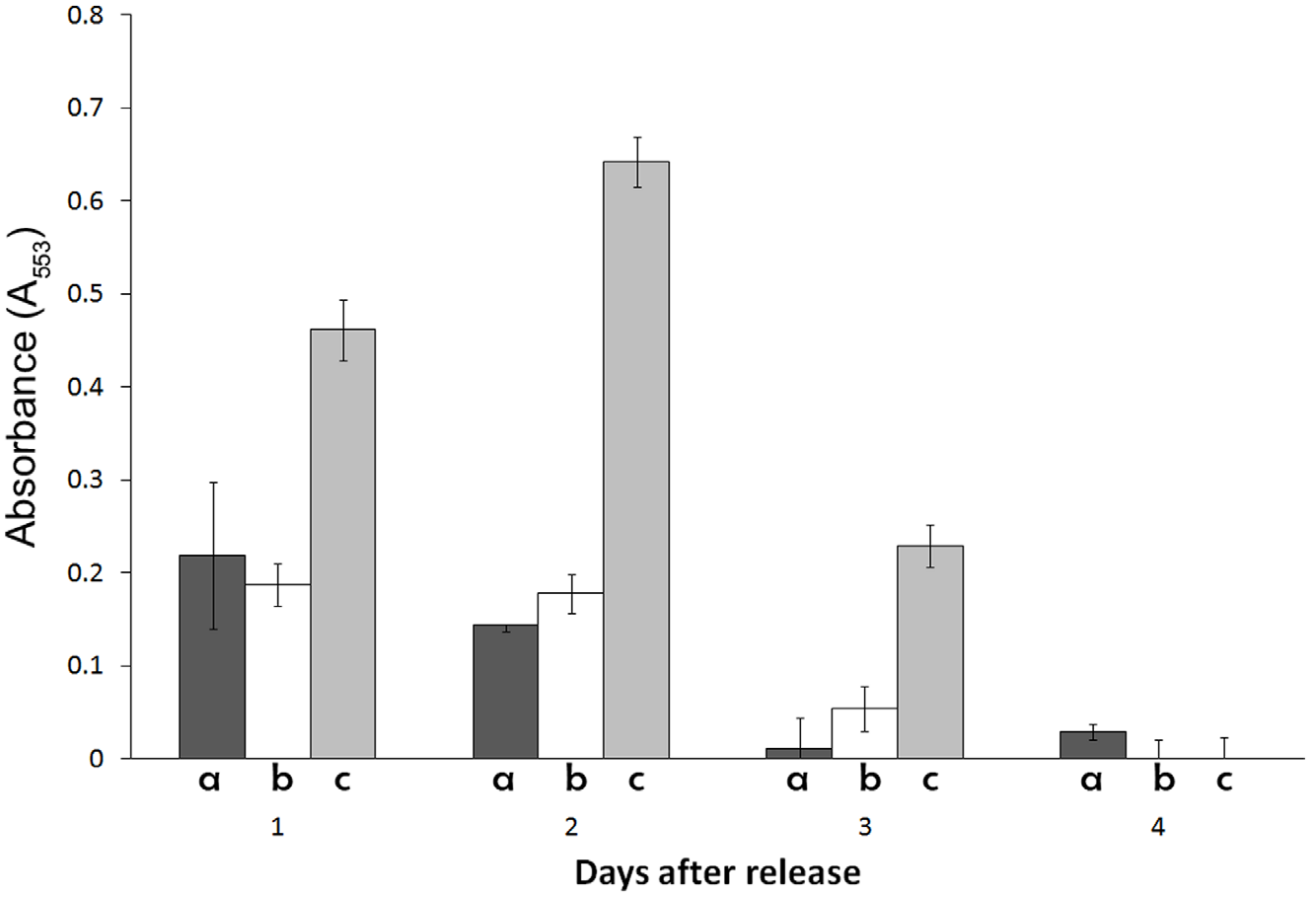
The elimination of p26 does not affect cyst metabolic activity. The metabolic activities of cysts lacking (a) and containing (b) p26, and of nauplii from commercially obtained cysts (c), were measured at daily intervals (1c4) as described in Methods. Absorbance (A_553_) in arbitrary units indicates the level of metabolic activity. The experiment was done in triplicate and absorbance values were averaged. Standard errors are shown for each measurement.

**Figure 4:**
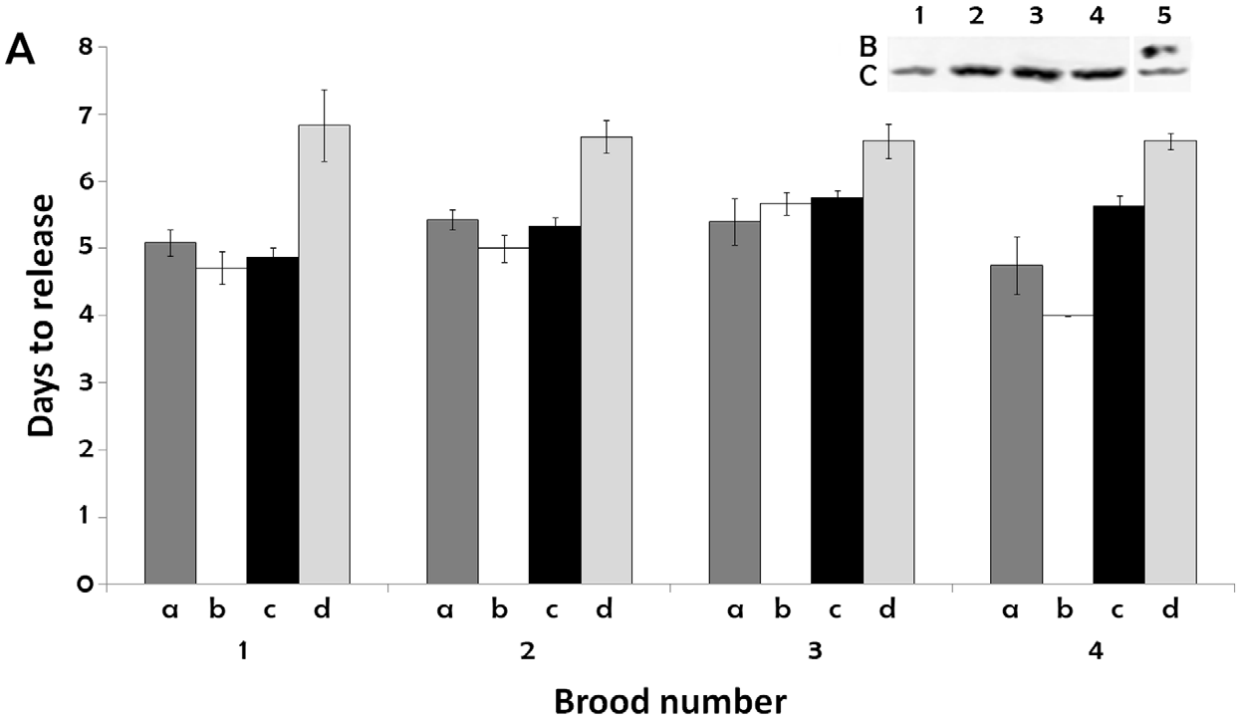
p26 knock down slows the development of diapause-destined *Artemia* embryos. A. The time to release in days for cysts and nauplii from females injected with control solution (a, c) and p26 dsRNA (b, d) for broods 1–4. a, nauplii; b, nauplii; c, cysts; d, cysts. n is 3 to 12 with lower values in higher brood numbers due to death of females with increased culture time. The bars indicate standard error for each measurement. Inset B. Protein extracts from cysts in broods 1–4 were resolved by SDS polyacrylamide gel electrophoresis, blotted to nitrocellulose and probed with antibody to p26 followed by HRP conjugated goat anti-rabbit IgG. Inset C. After stripping the blot was probed with antibody to ArHsp21. Lane 1, brood 1; 2, brood 2; 3, brood 3; 4, brood 4; 5, protein extract from commercially obtained cysts containing p26 and ArHsp21. Proteins reacting with antibodies were visualized by chemiluminescence.

### Detection of p26 mRNA by RT-PCR

Fifty hydrated *Artemia* cysts from females injected with either dsRNA or control solution were collected by centrifugation at 500 RPM in a microcentrifuge and suspended in 600 µl of RLT reagent from the RNAeasy kit (Quiagen, Mississauga, ON, Canada). The cysts were homogenized with a microfuge pestle (Fisher Scientific, Ottawa, ON, Canada), passed through a 20 gauge needle several times and centrifuged at 500 RPM for 1 min in a microcentrifuge. The resulting supernatants were transferred to fresh tubes prior to RNA isolation with the RNAeasy kit (Quiagen). cDNA was produced with Superscript III® reverse transcriptase (Invitrogen) and oligo-dT primers (Invitrogen) according to manufacturer’s instructions, except the transcriptase was refreshed after 1 h and incubation was extended for 1 h. cDNA encoding p26 was then amplified by PCR using p26-specific primers as described above except that 38 cycles were used. Amplification products were resolved by agarose gel electrophoresis as described and DNA was visualized with SYBR® Safe DNA gel stain (Invitrogen).

**Figure 5:**
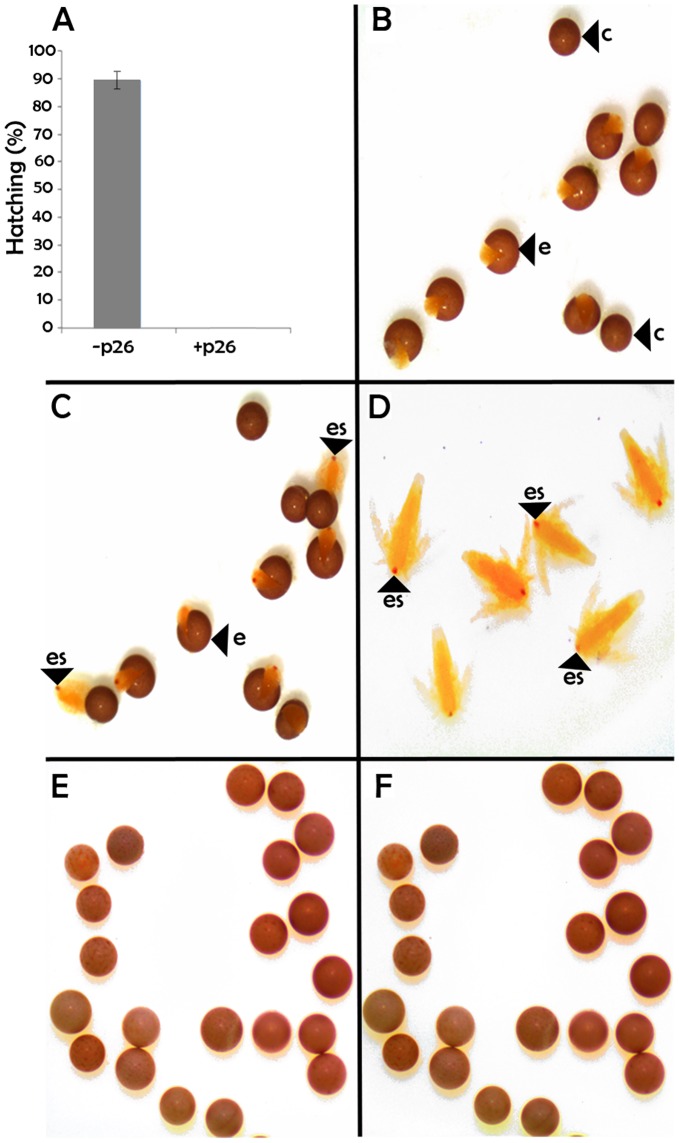
*Artemia* cysts lacking p26 terminate diapause spontaneously. A. Cysts lacking (−p26) and containing (+p26) p26 were incubated without agitation in sealed tubes for at least 90 days, transferred to weigh boats and observed with a dissecting microscope until 5 days after the last cyst hatched. The experiment was done in duplicate with different broods of cysts and the error bar represents standard error. B–F. Light micrographs showing the development, or absence thereof, for cysts lacking (B–D) and containing (E, F) p26. c, cyst; e, emerged cyst; es, eye spot. The nauplii shown in Fig. D originated from commercial cysts and they are representative of nauplii obtained from laboratory reared cysts lacking p26.

### Detection of sHSPs by SDS Polyacrylamide Gel Electrophoresis and Immunoprobing of Western Blots

To produce protein solutions for each lane of an SDS polyacrylamide gel 25 cysts from females injected with either p26 dsRNA, control solution or GFP dsRNA were collected by centrifugation for 1 min at 20 g in a microcentrifuge. Cysts were homogenized with a microfuge pestle in 2× treatment buffer (250 mM Tris, 280 mM SDS, 40% (v/v) glycerol, 0.2% (w/v) bromophenol blue, pH 6.8), placed in a boiling water bath for 5 min and centrifuged for 10 min at 8600 g in a microcentrifuge. Fifteen µl of each protein sample was resolved in 12.5% SDS polyacrylamide gels and either stained with Coomassie blue or transferred to nitrocellulose membranes (Bio-Rad, Mississauga, ON, Canada). Fermentas unstained and pre-stained plus protein ladders were used as molecular mass markers.

**Figure 6:**
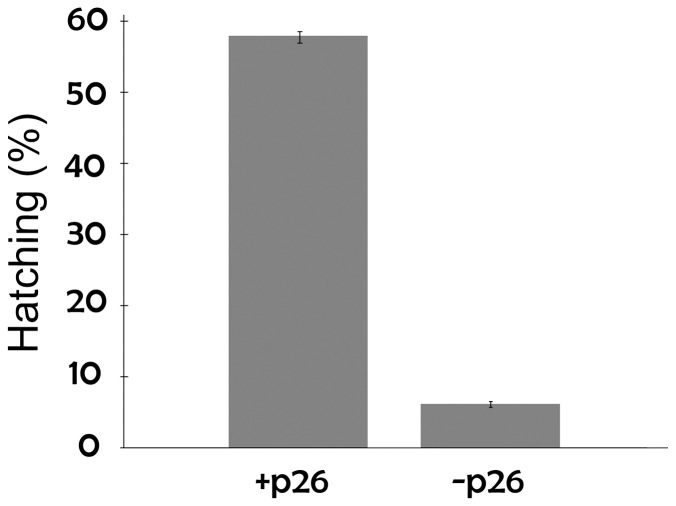
p26 enhances the stress tolerance of *Artemia* cysts. The diapause of *Artemia* cysts either containing (+p26) or lacking (−p26) p26 was terminated by desiccation and freezing. The cysts were then incubated in sea water at room temperature and hatched nauplii were counted and removed. The results are given as the percentage of cysts that hatched which was taken as the level of diapause termination and cyst viability. The experiment was done in triplicate with separate broods of cysts and the error bars represent standard error.

**Figure 7:**
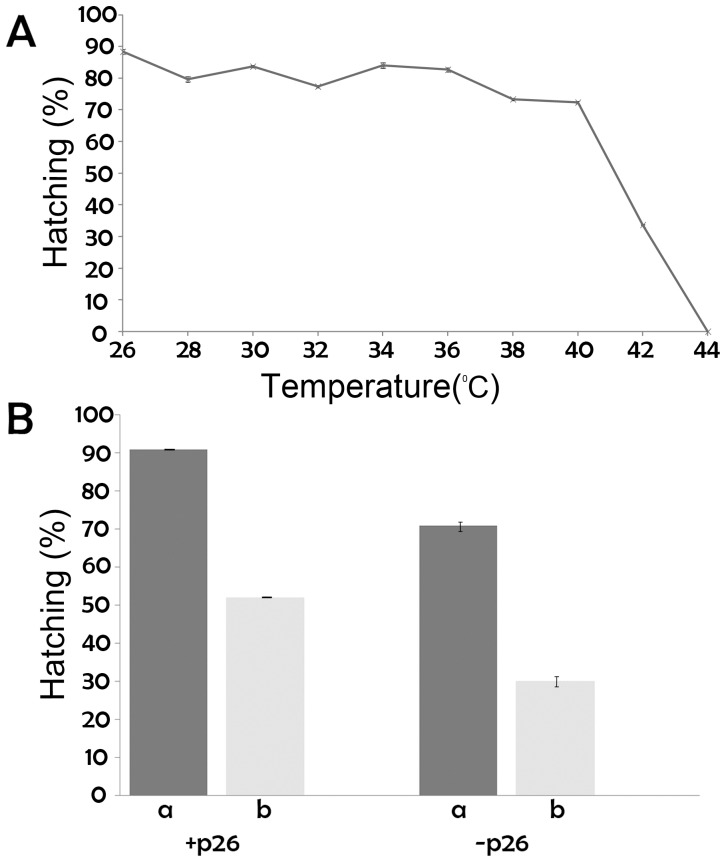
p26 increases cyst tolerance to heat shock. A. Hydrated commercial cysts containing p26 were heat shocked for 30 min at the temperatures indicated in the graph, cooled on ice, incubated in sea water at room temperature and observed for hatching. The experiment was done in triplicate and standard error is shown for each measurement. B. Cysts containing (+p26) and lacking (−p26) p26 were either held at room temperature (a) or heated at 41°C for 30 min (b), cooled on ice, incubated in sea water and observed for hatching. Results are presented as the hatching percentage with hatching equivalent to viability. The experiment was done in duplicate with separate broods of cysts and the error bars represent standard error.

Nitrocellulose membranes containing cyst proteins separated by electrophoresis were incubated in 5% Carnation low fat milk solution for 45 min at room temperature, then in antibody specific to p26 [37] diluted 1∶10000 in 10 mM TRIS containing 140 mM NaCl, pH 7.4 (TBS) for 20 min. The membranes were washed 3 times for 5 min in TBS containing 0.1% Tween 20 (TBS-Tween) and in 10 mM TRIS containing 1 M NaCl and 0.5% Tween 20, pH 7.4 (HST) 3 times for 5 min prior to incubation for 15 min in HRP-conjugated goat anti-rabbit IgG antibody (Sigma-Aldrich) diluted 1∶10000 in TBS. The membranes, washed as above, were rinsed in TBS and antibody-reactive proteins were visualized with ECL plus western blotting detection reagents (GE Healthcare, Baie d’Urfe, QC, Canada) followed by exposure to Bio-maxx® film (Kodak, Toronto, ON, Canada) or with a DNR Bio-Imaging Systems MF - ChemiBIS 3.2 gel documentation system (Montreal Biotech). ArHsp21 and ArHsp22 were identified on western blots by the same method except antibodies specific to these *Artemia* sHSPs [16], [17], respectively diluted at 1∶5000 and 1∶2000 in TBS, were employed.

### Assessment of Cyst Metabolic Activity

Metabolic activity was evaluated using an assay modified from Yang and Balcarcel [38] based on extracellular acidification of incubation medium. Ten cysts, either with or without p26, were incubated immediately after release from females in 100 µl of test solution consisting of seawater containing 1000 U penicillin, 100 µg/ml streptomycin sulfate and 0.03% phenol red at pH 8.5 in covered Costar 96 well UV plates (Corning Inc., Corning, NY, USA). Antibiotic concentrations were from Warner et al. [39]. Other wells contained test solution only or test solution with 10 commercially obtained cysts (INVE Aquaculture, Inc.) known to be metabolically active. The absorbance of test solutions at 553 nm (A_553_) was determined with a SPECTRAmax PLUS microplate reader (Molecular Devices, Sunnyvale, CA, USA) at 24 h intervals after addition of cysts to wells. To measure A_553_ the test solution from each well, excluding cysts, was transferred to a fresh 96 well plate. Absorbance values were corrected for changes that occurred in test solutions in the absence of added cysts. After A_553_ determination residual liquid was removed with autoclaved Q-tips from the original wells containing cysts and 100 µl of fresh test solution was added for the next 24 h incubation.

### Monitoring Embryo Development within *Artemia* Females

*Artemia* females destined to produce cysts or nauplii [1] were injected with either p26 dsRNA, control solution or dsRNA for GFP. Females mated in 6 well plates 48 hr after injection were monitored with an Olympus SZ61 stereomicroscope for fertilization, denoted by fusion of egg sacs, and to determine when cysts and nauplii were released post-fertilization (time to release). Twelve females were used in each experiment, although not all animals survived and the experiment was repeated several times. The time to release of successive broods of cysts was determined for several injected females in order to establish the longevity of the dsRNA effect. Protein extract prepared from each brood of cysts was resolved in SDS polyacrylamide gels, blotted to nitrocellulose and probed with antibody specific to p26 and ArHsp21.

### Diapause Termination and Cyst Hatching

Cysts released from *Artemia* females injected with either p26 dsRNA, control solution or GFP dsRNA were incubated for 90 days at room temperature in 10 ml of sea water in tightly capped 15 ml plastic tubes. The cysts were transferred to weigh boats by flushing the tubes with sea water and then observed under LED lighting (Intralux 4000, Volpi, Auburn, NY, USA) with an Olympus SZ61 stereomicroscope. Cyst development was documented with an Infinity 1-1 camera (Lumenera, Ottawa, Ontario, Canada). Sea water salt concentration was maintained by periodic addition of distilled water to weigh boats and experiments were terminated 5 days after the last cyst hatched. Nauplii were removed during incubation and non-developed cysts were decapsulated [12] at the end of the experiment to determine if they contained embryos. The experiment was done in duplicate.

### Determining the Stress Tolerance of *Artemia* Cysts

Diapause in laboratory produced p26-containing cysts was terminated by incubation at room temperature in sea water for 10 days after release from females, drying in a desiccator over DryRite (DryRite, Nashville, TN, USA) for 4 weeks and freezing at −20°C for at least 8 weeks. The extent of diapause termination was established by counting the number of cysts capable of hatching upon incubation in sea water at room temperature. Nauplii were removed as they hatched and experiments ended five days after hatching was last observed. The experiments were repeated with cysts lacking p26 to determine if the sHSP protected against stresses associated with the diapause termination procedure.

The contribution of p26 to stress tolerance was also determined by heat shocking *Artemia* cysts. To determine heat shock parameters microcentrifuge tubes with 100 commercially obtained hydrated cysts were incubated in a water bath for 30 min at defined temperatures. The cysts were cooled immediately on ice, transferred to 25 ml of sea water and incubated at room temperature. Hatched nauplii were counted and removed. The experiment was done in triplicate and counting ended 5 days after hatching was last observed. Once heat shock parameters were established commercially obtained cysts possessing p26 and laboratory produced cysts lacking p26 were either transferred to weigh boats without heating, or heated at 41°C for 30 min and then placed in weigh boats for incubation at room temperature. Nauplii were counted and removed and experiments ended five days after hatching was last observed. The experiment was done in duplicate with cysts from different females.

## Results

### Injection of *Artemia* Females with dsRNA

p26 dsRNA used for injection of *Artemia* females migrated in agarose gels at the expected size (Figure 1A). Prior to fertilization female *Artemia* destined to produce cysts possessed greenish oocytes positioned in two egg sacs separated by a brown shell gland (Figures 1B, C). Injection of an egg sac with either p26 dsRNA or control solution, both containing phenol red, stained the entire female, demonstrating that inoculum migrated readily throughout the animal (Figure 1D). Approximately 90% of females exhibited phenol red staining 2 h post-injection (Figure 1E) but the dye disappeared by the next day. Only animals maintaining dye for at least 2 h and exhibiting normal behavior after injection were used in experiments. Appendages normally visible on adult *Artemia* and poorly resolved in the upper portion of Figure 1C are obscured in the lower magnification Figures 1B, D, E because animals were immobilized on the surface of cold agarose and both animals and agarose were partially dried with a Kimwipe prior to injection.

### Injection of *Artemia* Females with p26 dsRNA Specifically Knocked Down p26 mRNA and Protein in Cysts

Electrophoresis of RT-PCR products yielded a strong band in agarose gels stained with SYBR® Safe DNA when mRNA from cysts produced by *Artemia* females injected with control solution was used for amplification (Figure 2A, lane 1). When mRNA was from cysts generated in females injected with p26 dsRNA the PCR product was greatly diminished, demonstrating the reduction of p26 mRNA (Figure 2A, lane 2). As revealed by immunoprobing of western blots, p26 occurred in cysts released from females injected with control solution, but the protein was not detectable in cysts from females receiving p26 dsRNA even when blots were over-exposed (Figure 2B). Additionally, injection of *Artemia* females with dsRNA for GFP had no effect on the quantity of p26 protein in cysts (not shown). ArHsp21 and ArHsp22, sHSPs similar to p26 and synthesized in diapause-destined *Artemia* embryos, were unaffected by the injection of females with p26 dsRNA (Figures 2C, D) further demonstrating the specificity of the knock down procedure.

### The Metabolic Activities of Normal and p26 Knock Down Cysts were Similar

The metabolic activities of cysts newly released from females were very similar to one another whether or not the cysts contained p26 (Figure 3, bars a, b). Metabolism declined, usually to levels non-detectable by the assay, 3–4 days after release. Examination of nauplii hatched from commercial cysts was performed to demonstrate that metabolic activity was detectable at all stages of the assay developed and employed herein (Figure 3, bar c). The metabolic activity of nauplii decreased by 3 days post-hatching because they were not fed.

### p26 Knock Down Selectively Slowed the Development of Diapause-destined *Artemia* Embryos

The time from fertilization to release of either cysts or nauplii (time to release) was approximately 5 days for first broods from females injected with control solution; this is comparable to the time to release of nauplii from females injected with p26 dsRNA (Figure 4) and for nauplii and cysts from non-injected females and females injected with dsRNA for GFP (not shown). In contrast, injection of females with p26 dsRNA increased the time to release for first brood cysts to approximately 7 days (Figure 4). The developmental delay imposed by elimination of p26 persisted for at least 3 additional broods, the maximum number observed (Figure 4). No p26 was detectable by immunoprobing of western blots containing protein from cysts experiencing developmental delays although stripping of blots and probing with antibody to ArHsp21 gave strong bands (inset, Figures 4B, C). Delays in embryo development did not carry over to animals arising from p26-free cysts and these subsequent generations produced cysts with normal amounts of p26 (not shown).

### Cysts Lacking p26 Terminated Diapause Spontaneously

After incubation in sea water at room temperature for at least 90 days approximately 90% of cysts lacking p26 hatched upon transfer to weigh boats, indicating diapause termination (Figures 5A–D). Under the same experimental conditions cysts containing p26 failed to hatch (Figures 5A, E, F). The results indicate that p26 prevents diapause termination until an appropriate signal is received by cysts.

### p26 Protected *Artemia* Cysts against Stress

Diapause termination in laboratory produced cysts required exposure to stressful conditions including 4 weeks of desiccation followed by 8 weeks at −20°C. Hatching success varied and 58% of laboratory produced cysts containing p26 survived diapause termination and hatched as opposed to only 6% of cysts lacking p26 (Figure 6). These data indicate that p26 contributes to stress protection and to test this idea, cysts were heat shocked. Heating of p26-containing cysts (INVE Aquaculture, Inc.) for 30 min over a range of temperatures established that 56% of cysts survived exposure to 41°C (Figure 7A) and these conditions were chosen for subsequent heat shock experiments. p26-containing commercial cysts and laboratory reared cysts lacking p26 which terminate diapause without desiccation and freezing (see Figure 6), were transferred to weigh boats before and after heating at 41°C for 30 min. For p26 containing cysts 91% and 52% respectively of unheated and heated cysts hatched whereas when p26 was absent 71% of unheated and 30% of heated cysts hatched (Figure 7B). Cysts from different sources were compared because terminating diapause in laboratory produced cysts required exposure to stress (Figure 6). The findings demonstrate that p26 increases tolerance to heat shock.

## Discussion

Molecular chaperones up-regulated during diapause are thought to enhance stress resistance [3], but evidence supporting this proposal, although strong, is mostly correlative. For example, *Artemia* cysts, one of the most stress tolerant life forms known among animals, accumulate at least three sHSPs, with p26 in abundance [1], [13], [15]–[17], as well as other molecular chaperones such as artemin [18], [19]. sHSPs are abundant in resting eggs of the rotifer *Brachionus plicatilis* [40], [41] and the sHSP Hsp22 increases during the diapause of *Calanus finmarchicus*, a marine copepod [42]. Stress tolerant pupae of the flesh fly, *S. crassipalpis*, up-regulate several sHSP family members when in diapause [43] whereas the fruit fly *D. triaura*, with minimal metabolic depression during diapause, does not increase sHSPs [44], [45]. The protective properties of only a few diapause-specific sHSPs, including those from *A. franciscana* [1], [16], [17] and *S. crassipalpis* [43] have been characterized by *in vitro* experiments and RNAi knock down. In addition, the effects of RNAi mediated reduction of forkhead transcription factor (FOXO), catalase and superoxide dismutase-2, the latter two proteins involved in reactive oxygen species (ROS) detoxification, have been examined during diapause in the mosquito *Culex pipiens* [46], [47].

p26, the diapause-specific sHSP found in *Artemia* cysts, functions as a molecular chaperone, preventing heat- and reduction-induced protein aggregation in vitro [14], [34], and increasing stress tolerance when synthesized in either transformed bacteria [34], [25] or transfected mammalian cells [29], [35]. p26 inhibits apoptosis in mammalian cells, a property shared with other sHSPs [29], [35], [48] but p26 activity had not been tested previously in vivo. To this end, RNAi methodology was used to knock down p26 in diapause-destined *Artemia* embryos. RNAi has been employed in *Artemia* to show *caudal* gene function in axis elongation and segmentation [36] and the repression of Hox genes by *spalt* [49]. A role for p90 ribosomal S6 kinase in the termination of cell cycle arrest as post-diapause *Artemia* embryos resume development is indicated by the use of RNAi [50]. The requirement for shell gland-specifically expressed genes SGEG1 (previously SGEG) and SGEG2 as integral components of stress resistance attributed to the *Artemia* cyst shell [51], [52] and the role of cyclin K in development of diapause embryos have been examined by RNAi [53]. The work described in this paper demonstrated that p26 dsRNA injected into the egg sacs of *Artemia* females crossed oocyte membranes affecting embryo development, diapause maintenance/termination and stress tolerance but not metabolic activity. Transduction of dsRNA from females into embryos occurs in the flour beetle, *Tribolium castaneum* [54], but similar observations have not been widely reported for arthropods.

Injection of p26 dsRNA into *Artemia* females partially knocked down p26 mRNA in cysts, a reduction in transcripts similar to that shown for *caudal* mRNA upon injection of *Artemia* with *caudal* dsRNA [36] and for catalase mRNA when *C. pipiens* is injected with catalase dsRNA [47]. p26 protein decreased to such an extent that it was not detectable upon immunoprobing of western blots, even upon over-exposure and when p26 in extracts of cysts from females injected with control solution and dsRNA for GFP reacted strongly with antibody. The knock down was further shown to be specific because ArHsp21 and ArHsp22, *Artemia* sHSPs produced in diapause-destined embryos and sharing a conserved α-crystallin domain with p26, were not reduced upon injection of females with p26 dsRNA. In complementary experiments, injection of females with dsRNA for ArHsp21 and ArHsp22 had no effect on p26 accumulation in cysts (manuscript in preparation). These results show that changes in cysts upon injection of females with p26 dsRNA were due to diminished p26. The RNAi effect was long lasting with the increase in time to release and the absence of p26 in cysts persisting until at least the fourth brood obtained from females injected once with p26 dsRNA. However, the increase in time to release and the reduction of p26 were not apparent for cysts produced by females originating from p26-free cysts, demonstrating that the RNAi effect was not transferred to new generations.

The increase in time to release of cysts from females injected with p26 dsRNA versus GFP dsRNA or control solution revealed that the loss of p26 slowed the development of diapause-destined embryos or, in other words, p26 is required to attain the maximum rate of development of diapause-destined embryos. How this occurs has yet to be determined. These data suggest that p26 does not arrest DNA replication and the cell cycle during diapause, as proposed for sHSPs in other organisms where their accumulation correlates with reduced growth [55], [56]. That another sHSP such as ArHsp21 or ArHsp22 [16], [17] impedes DNA replication and/or cell division in diapause-destined *Artemia* embryos is possible, but preliminary evidence suggests otherwise. Equally interesting is the observation that p26 prevents diapause termination in cysts after long term incubation in sea water. The molecular mechanism explaining this result is unclear but may occur either because p26 sustains maintenance by binding proteins critical to the regulation of this phase of diapause, or sequesters signaling proteins important for diapause termination. p26 may also prevent diapause termination by countering the effects of reactive oxygen species (ROS) [3], [12].

Diapause was terminated in p26-containing cysts by desiccation followed by freezing and thawing. Drying and cold contribute to the termination of *A. franciscana* diapause in natural environments [57] and they have been used by others to terminate diapause in the laboratory [51]. Although desiccation and freezing are stressors diapause termination was higher at 3 months, as opposed to earlier times, but it increased only marginally from 3 to 8 months (not shown). Cysts with and without p26 exhibited equivalent metabolic activity upon release from females, but hatching was greatly reduced upon diapause termination induced by desiccation and cold in cysts lacking p26 as opposed to cysts containing this sHSP. These findings, along with the observation that p26 increases heat tolerance indicate that this sHSP contributes to stress tolerance during diapause, probably by protecting proteins from irreversible denaturation. Clearly, p26 affects development, diapause termination and stress tolerance in diapause embryos. Mechanistic similarities between these activities may centre on the ability of p26 to bind proteins, either to inhibit potentially damaging activities such as apoptosis or prevent their irreversible denaturation. Roles in cyst development, diapause termination and protection against stress reflect the presence of p26 in both the cytoplasm and nucleus [10], [13], [14], [20], [24], [33].

In conclusion, the results in this paper show that p26 contributes to stress tolerance during diapause in *Artemia* embryos, and it reveals that p26 modulates diapause-destined embryo development and diapause termination. The work therefore contributes to our fundamental understanding of sHSP function during diapause, an important developmental/physiological process found in many animals and especially in insects. There are also practical implications. For example, *Artemia* is a common feed source for commercially important species and understanding diapause will have applications in aquaculture. Moreover, because diapause is common in many insect pests, elucidating the molecular mechanisms of this process has practical implications in forestry, agriculture and medicine.
